# Artificial Intelligence Algorithm-Based Intraoperative Magnetic Resonance Navigation for Glioma Resection

**DOI:** 10.1155/2022/4147970

**Published:** 2022-03-04

**Authors:** Jianqiang Wei, Chunman Zhang, Liujia Ma, Chunrui Zhang

**Affiliations:** ^1^Neurovascular Interventional Therapy Center, Affiliated Hospital of Yan'an University, Yan'an 716000, Shaanxi, China; ^2^Department of Neurosurgery, Affiliated Hospital of Yan'an University, Yan'an 716000, Shaanxi, China; ^3^Department of Neurology, Hanzhong People's Hospital, Hanzhong 723000, Shaanxi, China

## Abstract

The study aimed to analyze the application value of artificial intelligence algorithm-based intraoperative magnetic resonance imaging (iMRI) in neurosurgical glioma resection. 108 patients with glioma in a hospital were selected and divided into the experimental group (intraoperative magnetic resonance assisted glioma resection) and the control group (conventional surgical experience resection), with 54 patients in each group. After the resection, the tumor resection rate, NIHSS (National Institute of Health Stroke Scale) score, Karnofsky score, and postoperative intracranial infection were calculated in the two groups. The results revealed that the average tumor resection rate in the experimental group was significantly higher than that in the control group (*P* < 0.05). There was no significant difference in Karnofsky score before and after the operation in the experimental group (*P* > 0.05). There was no significant difference in NIHSS score between the experimental group and the control group after resection (*P* > 0.05). The number of patients with postoperative neurological deficits in the experimental group was smaller than that in the control group. In addition, there was no significant difference in infection rates between the two groups after glioma resection (*P* > 0.05). In summary, intraoperative magnetic resonance navigation on the basis of a segmentation dictionary learning algorithm has great clinical value in neurosurgical glioma resection. It can maximize the removal of tumors and ensure the integrity of neurological function while avoiding an increased risk of postoperative infection, which is of great significance for the treatment of glioma.

## 1. Introduction

Glioma is one of the most common diseases in neurosurgery, which belongs to malignant tumors. Glioma plays an important role in intracranial tumors. About half of intracranial tumors are gliomas clinically [1]. In recent years, the incidence of glioma has been increasing. According to statistics, there are up to 15,000 new glioma patients in the United States every year. The incidence of intracranial tumors in China is about 0.1%. Nearly half of the newly diagnosed patients were diagnosed with glioma [2–4]. The cancerous glial cells in glioma patients are derived from the neuroectodermal layer. Not only is the incidence higher than other intracranial tumors, but also the recurrence rate is very high after various treatments. Therefore, compared with other intracranial tumors, glioma has the characteristics of high mortality and a low cure rate [5–7]. In China, gliomas are divided into eight categories, among which astrocytoma has the highest incidence. Clinically, gliomas are divided into four grades according to the degree of malignancy of astrocytomas. Grade I is relatively mild. It is mainly manifested as hair cell astrocytoma, which accounts for about 5% of the incidence of glioma. Benign tumors are often diagnosed and can be cured by resection in theory. Grade II is more serious; mainly as astrocytoma and a small amount of astrocytoma, accounting for about 35% of glioma. Grade III is mostly developed from grade II glioma. Interstitial astrocytomas accounted for about 15–25% of gliomas. The disease is serious and highly malignant. Grade IV is the most serious malignancy, accounting for one third of all gliomas. It is mainly manifested as glioblastoma [8–12].

With the rapid development of science and technology and the improvement of medical level, more and more treatment methods for glioma are appearing in people's eyes. Drug therapy as one of the treatment methods has achieved good results. The selection of appropriate chemotherapy drugs according to the specificity of glioma can inhibit the growth of tumors and delay or limit the deterioration of tumors to a certain extent [13–15]. The disadvantages of chemical drug therapy are the side effects of drugs, which limit the development and application of drug therapy. Radiotherapy also plays a certain role in the clinical treatment of glioma. Radiotherapy can kill radiation-sensitive tumor cells and limit the development of tumors. In addition, radiotherapy has great advantages for deep tumors that cannot be reached by surgery [16–19]. The dose of the first two treatment methods needs to be strictly controlled, and there will be some damage to normal tissues, which is greatly limited in the treatment of glioma [20]. Therefore, surgical treatment is the most commonly used and effective treatment of glioma in clinics [21]. Gliomas with a low degree of deterioration are mostly cured by surgery, while gliomas with a high degree of deterioration can greatly prolong the life span of patients by surgical treatment. Glioma resection is the most critical in the treatment of glioma [22–25]. The resection can minimize the tumor volume, reduce the number of tumor cells, reduce intracranial hypertension and prolong the life of patients [26].

Magnetic resonance imaging (MRI) plays an important role in neurosurgical treatment and diagnosis. Intraoperative magnetic resonance imaging (iMRI) appeared in the late twentieth century [27]. MRI has a high resolution on the soft tissue of the body, which plays an important role in the diagnosis of glioma and postoperative review [28]. At present, iMRI technology can provide real-time imaging information during glioma surgery, which has a great role in promoting glioma resection and makes neurosurgical glioma resection enter a new period [29–31].

In this study, 108 patients with glioma were collected as research subjects, and two different methods were used to conduct experiments during surgery to analyze the application value of iMRI based on artificial intelligence algorithms in neurosurgical glioma resection.

## 2. Materials and Methods

### 2.1. Research Objects

This study selected 108 patients with glioma in hospitals from February 2, 2018 to June 2, 2021. Among them, there were 58 males and 50 females, ranging in age from 27 to 65 years old, with an average age of 40.28 ± 3.76 years old. The patients were randomly divided into an experimental group (patients with iMRI-assisted glioma resection) and a control group (patients with conventional surgical resection), with 54 cases in each group. All the subjects agreed to sign informed consent forms with the consent of their family members, and this study had been approved by the ethics committee of the hospital.

Inclusion criteria were as follows: (1) clinical symptoms and laboratory tests have been diagnosed with glioma patients; (2) no severe heart, lung, or abdominal diseases; (3) no other intervention measures were implemented before the operation; and (4) patients without any examination of contraindications.

The exclusion criteria were as follows: (1) critically ill patients; (2) patients with severe cardiopulmonary or abdominal diseases; (3) patients unable to cooperate with surgical treatment due to mental illness; (4) patients whose family members did not agree and did not sign the informed consent; and (5) older patients (older than 70 years) or patients who cannot undergo craniotomy.

### 2.2. Intraoperative Magnetic Resonance-Guided Neurosurgical Glioma Resection

**Figure 1:**
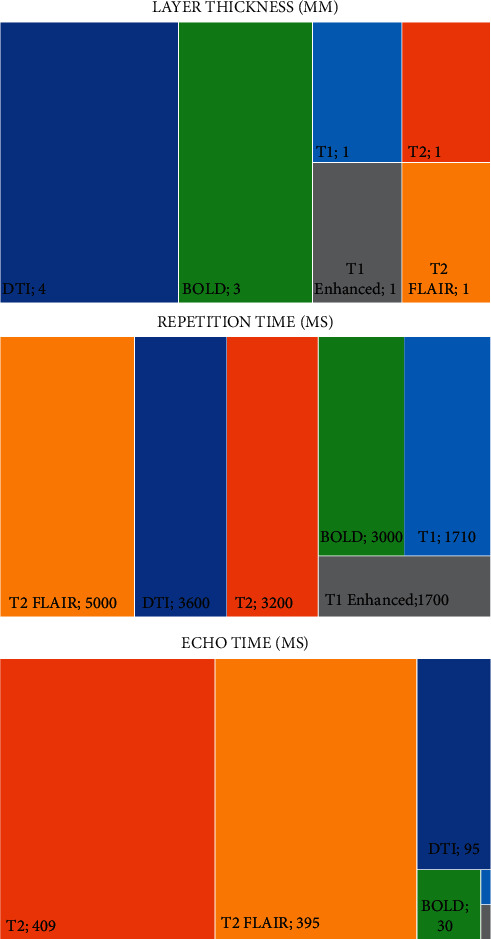
The parameters of MRI scanning sequence during operation.

Before surgery, the patients were scanned using magnetic resonance technology to obtain imaging data, determine the regional scope, boundary, and nerve conduction bundle in the adjacent region of glioma, and prepare for real-time navigation during surgery. The specific scanning sequence parameters are shown in Figure 1.

The glioma patients in the experimental group were prepared strictly according to the intraoperative magnetic resonance scanning navigation standard. First, patients need to wear earplugs. Then, it is important to ensure there is no skin contact between the upper and lower limbs, palms and fingers, and armpits. All kinds of instrument accessories including monitor and catheter, magnetic resonance coil wire, and arteriovenous infusion tube did not have any contact with the skin. Ensure that there is no metal in the surgical area. The patient is placed in the right position as required. The head is fixed, and the navigation frame is installed to ensure that the head area can be detected by the instrument. Patients were routinely disinfected, spread sheets were placed, skin incisions were made, and skull drilling was carried out after craniotomy. The doctor determined the surgical plan according to the navigation image and performed glioma resection. During iMRI scanning, the surgical area was covered with sterile gauze and then wrapped with a sterile cover. During the operation, magnetic resonance professionals were asked to verify the work, including patients during the operation, air and ground objects in the safe area, and all safety measures during the operation were closed. The screen door was opened, and intraoperative magnetic resonance scanning was performed after the magnet was removed to check the tumor resection at any time. The regional boundary of residual glioma was marked by neuronavigation, and the resection plan was formulated, which was completely resected under neuronavigation. At the same time, in order not to damage other functional areas, irreversible damage to nerve function was avoided.

### 2.3. Segmentation Dictionary Learning Algorithm

**Figure 2:**
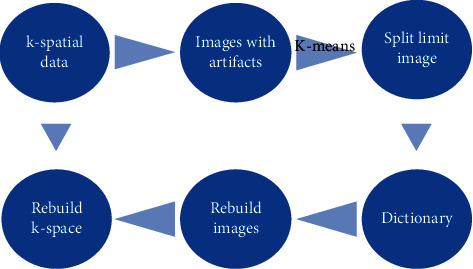
Flow chart of the segmentation dictionary learning algorithm.

A segmented dictionary learning algorithm can overcome the problem of small signal and large differences between tissues in MRI reconstruction images. Before reconstruction, the image is segmented according to the characteristics, and the initial image classification is obtained based on the image gray level. The same type of organization uses a unified image gray mean to constrain, and then the image with the segmentation constraint is reconstructed to construct the dictionary. The specific process is shown in Figure 2.

### 2.4. Measurement of Glioma Resection

The glioma boundary was drawn under the enhanced MRI image, and the tumor volume before and after resection was obtained to calculate the tumor volume. The extent of glioma resection can be evaluated by tumor resection rate. The tumor resection rate was expressed as *T*, the preoperative glioma volume was expressed as *F*, and the postoperative tumor volume was expressed as *L*. The method of calculating the tumor resection rate was as follows:

(1) $$\begin{array}{c} T=\frac{F-L}{F}\times 100\%. \end{array}$$

According to the calculated glioma resection rate, the degree of tumor resection is divided into four grades: total resection, near-complete resection, secondary resection, and partial resection. The corresponding tumor resection rates were 100%, 90%–99%, 50%–89%, and <50%, respectively.

### 2.5. Assessment of Neurological Function in Patients with Glioma

Before and after glioma resection, Karnofsky function status scores were performed on the patients. The higher the score was, the better the health status of the patients was. The patients had a strong tolerance for the side effects of surgical treatment and a greater probability of complete cure. The lower the score, the worse the patient's physical condition. Once the score is lower than 60, a variety of antitumor treatments are difficult to implement.

Two days after glioma resection, the NIHSS score was measured. They were evaluated and recorded from the aspects of consciousness level, gaze, vision, facial paralysis, upper limb movement, lower limb movement, ataxia, sensation, language, dysarthria, and neglect. The lower the score, the better the patient's health, the less neurological damage.

### 2.6. Evaluation of Postoperative Infection in Glioma Patients

The evaluation of postoperative infection in patients with glioma was based on fever and cerebrospinal fluid examination results. Specific criteria are as follows: patients with postoperative fever, vomiting, headache, and meningeal irritation sign positive symptoms. The results of cerebrospinal fluid examination showed that white blood cells > 10^6^/L, while white blood cells > 10^10^/L in peripheral blood. The results of cerebrospinal fluid specimen examination showed that the sugar content was 0.45 g/L. The results of bacterial culture of cerebrospinal fluid or intracranial drainage tube showed positive.

### 2.7. Statistical Method

SPSS software was used for the statistical analysis of the data. The data in line with normal distribution were expressed as mean ± standard deviation. The *t* test was used to represent the measurement data, chi-square (*χ*^2^) test was used to represent the count data, and *P* < 0.05 meant the difference was statistically significant.

## 3. Results

### 3.1. MRI Results

**Figure 3:**
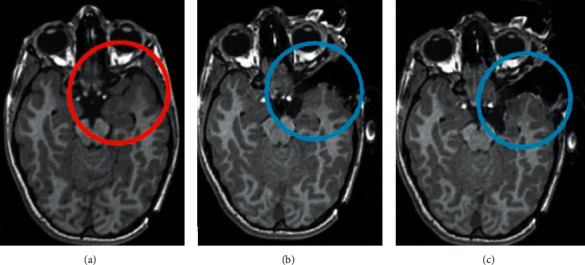
MRI of a patient with glioma before (a), during (b), and after (c) resection.

Figure 3 is a glioma patient with clinical manifestations of hyperactivity and low speech ability, and often seizures. Preoperative MRI showed a glioma located in the left temporal lobe, and neurosurgical glioma resection was performed under intraoperative magnetic resonance navigation based on a segmentation dictionary learning algorithm. Intraoperative magnetic resonance imaging revealed that the glioma was being cut, and there were some residual tumors. At the end of the operation, magnetic resonance imaging showed that the glioma had been completely resected.

**Figure 4:**
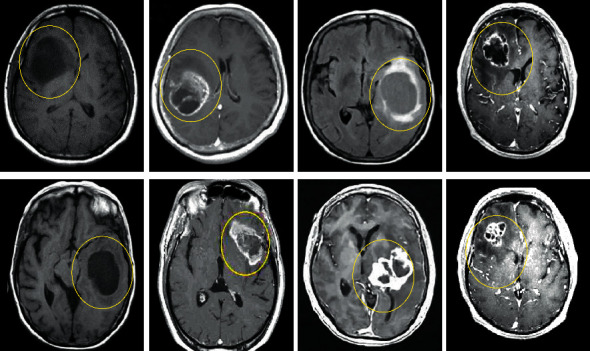
Magnetic resonance imaging results of glioma patients.

Figure 4 shows MRI images of different glioma patients. Gliomas distributed in different parts of the brain are shown in Figure 4, where the yellow circle indicates the location of a glioma.

### 3.2. Results of Glioma Resection Degree

**Figure 5:**
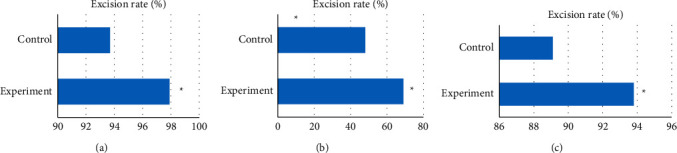
Comparison of tumor resection rate between the two groups. (a) The comparison of the average tumor resection rates of the two groups. (b) The comparison of the total tumor resection rates of the two groups. (c) The comparison of the subtotal tumor resection rates of the two groups. ^*∗*^significant difference: *P* < 0.05.

The degree of glioma resection was compared between the two groups by calculating the tumor resection rate of the experimental group and the control group. The results showed that the average tumor resection rate of the experimental group was significantly higher than that of the control group (*P* < 0.05). In addition, the resection rate in the control group was significantly lower than that in the experimental group (*P* < 0.05). The specific results are shown in Figure 5.

### 3.3. Neurological Function Score of Glioma Resection Patients

**Figure 6:**
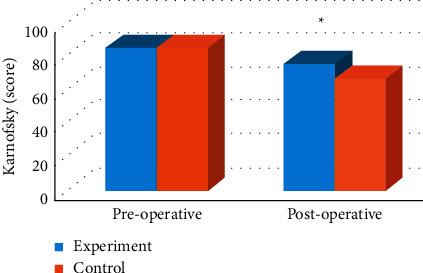
Comparison of Karnofsky scores between the two groups before and after glioma. Note: ^*∗*^significant difference, *P* < 0.05.

Karnofsky scores were compared between the two groups before and after surgery, and the results showed that there was no significant difference in Karnofsky scores before and after surgery in the experimental group (*P* > 0.05). The Karnofsky score in the control group was significantly lower than that before operation (*P* < 0.05). There was no significant difference in preoperative Karnofsky score between the two groups (*P* > 0.05), but there was a significant difference in postoperative Karnofsky score between the two groups (*P* < 0.05). The specific results are shown in Figure 6.

**Figure 7:**
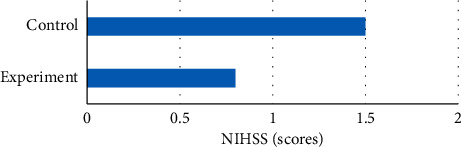
Comparison of NIHSS scores between the two groups after glioma resection.

**Figure 8:**
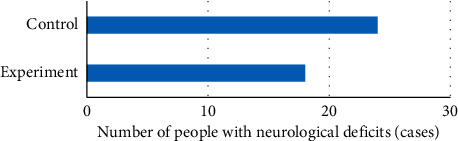
Comparison of neurological deficits between the two groups after glioma resection.

NIHSS was used to evaluate the postoperative neurological function of the two groups, and the results showed that there was no significant difference in NIHSS score between the experimental group and the control group (*P* > 0.05). In addition, the number of patients with different degrees of neurological deficit in the control group was close to half (24/54), and the number of patients with neurological deficit in the experimental group was less than that in the control group. The specific results are shown in Figures 7 and 8.

### 3.4. Evaluation Results of Postoperative Infection in Glioma Patients after Resection

**Figure 9:**
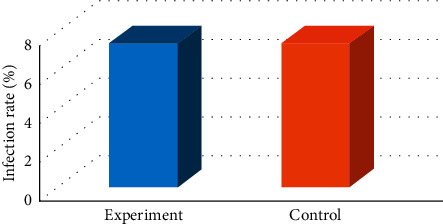
Comparison of infection rates between the two groups after glioma resection.

The postoperative infection rates of the two groups were evaluated, and it was found that the postoperative infection rates of glioma patients in the experimental group and the control group were 7.4% (4/54). There was no significant difference in infection rate (*P* > 0.05). The results are shown in Figure 9.

## 4. Discussion

At present, the incidence and mortality of glioma are increasing. Gliomas play an important role in intracranial tumors, and about half of the intracranial tumor clinics are gliomas [32]. The effective treatment of glioma has become a hot research direction for researchers in China and abroad. The treatment of glioma needs to be determined based on the specific circumstances of each patient. It is extremely individualized treatment. The main treatment principle is surgery, supplemented by other treatments. Therefore, a smooth and effective operation is a prerequisite for the treatment of this disease. In this study, intraoperative magnetic resonance navigation on the basis of an artificial intelligence algorithm is applied to neurosurgical glioma resection, and the clinical application value of intraoperative magnetic resonance navigation in neurosurgical glioma resection is studied. A total of 108 patients with glioma were selected and divided into the experimental group (patients with intraoperative magnetic resonance assisted glioma resection) and the control group (patients with conventional surgical experience resection), with 54 in each group. In the experimental group, the tumor resection rate, NIHSS score, Karnofsky score, and postoperative intracranial infection were calculated by real-time reconstruction of intraoperative magnetic resonance images on the basis of a segmentation dictionary learning algorithm. To evaluate the application value of intraoperative magnetic resonance navigation on the basis of segmentation dictionary learning algorithm in neurosurgical glioma resection.

The tumor resection rate is crucial to evaluate the success of glioma resection. The decrease of resection rate indicates that the recurrence rate of tumor will be significantly reduced and the life expectancy of patients will be prolonged. The results of this study showed that the average tumor resection rate in the experimental group was significantly higher than that in the control group (*P* < 0.05). This shows that under the guidance of intraoperative magnetic resonance navigation, glioma resection can better achieve tumor resection, which is of great significance to other adjuvant therapies for patients. The complete neurological function of patients after glioma resection is also an important indicator for evaluating surgery. In this study, Karnofsky scores were compared between the two groups of patients before and after surgery, and the results showed that there was no significant difference in Karnofsky scores before and after surgery in the experimental group (*P* > 0.05). NIHSS was used to evaluate the postoperative neurological function of the two groups, and the results showed that there was no significant difference in NIHSS score between the experimental group and the control group (*P* > 0.05). The number of patients with postoperative neurological deficits in the experimental group was smaller than that in the control group. This shows that under the guidance of intraoperative magnetic resonance navigation, the neurological function of patients after resection is relatively complete, which greatly guarantees the quality of life of patients after operation. In addition, the postoperative infection of the two groups was evaluated, and it was found that there was no significant difference in the infection rate between the patients with glioma guided by intraoperative magnetic resonance and the patients with conventional glioma resection (*P* > 0.05). Studies have found that intraoperative magnetic resonance scanning technology in glioma resection can significantly improve the tumor resection rate. Under the guidance of neural function navigation, real-time brain tissue functional images can be provided for the tumor resection process, and the resection area can be observed at any time to avoid damage to the neural function area or conduction bundle. Then, the most complete resection and the safest resection were achieved, which is consistent with the results of this study [33]. At the same time, the application of intraoperative MRI in tumor resection in this study did not increase the risk of postoperative infection. Therefore, the application of intraoperative MRI in glioma resection has great clinical value.

## 5. Conclusions

Intraoperative magnetic resonance navigation on the basis of an artificial intelligence algorithm was applied to neurosurgery glioma resection to study the clinical application value of intraoperative magnetic resonance navigation in neurosurgery glioma resection. A total of 108 patients with glioma were selected and divided into the experimental group (patients with intraoperative magnetic resonance assisted glioma resection) and the control group (patients with conventional surgical experience resection), with 54 in each group. In the experimental group, the tumor resection rate, NIHSS score, Karnofsky score, and postoperative intracranial infection were calculated by real-time reconstruction of intraoperative magnetic resonance images on the basis of a segmentation dictionary learning algorithm. The results revealed that under the guidance of intraoperative magnetic resonance navigation, glioma resection can better achieve tumor resection. In addition, the neurological function of patients after resection is relatively complete, which greatly ensures the quality of life of patients after operation. In addition, intraoperative MRI did not increase the risk of postoperative infection in patients undergoing tumor resection. Intraoperative magnetic resonance navigation on the basis of a segmentation dictionary learning algorithm has great application value in neurosurgery glioma resection. The deficiency of this study is that the sample size of the research object is too small, and the source is single, not random and widely applicable. In the subsequent studies, the analysis and research of multisite, multitype, and large sample sizes will be considered to provide a more practical and effective reference value for the application of intraoperative magnetic resonance navigation in neurosurgery glioma resection.

## Data Availability

The data used to support the findings of this study are available from the corresponding author upon request.

## References

[1] Deland K., Mercer J. S., Crabtree D. M. (2022). Radiosensitizing the vasculature of primary brainstem gliomas fails to improve tumor response to radiation therapy. *International Journal of Radiation Oncology, Biology, Physics*.

[2] Luo H., Tao C., Long X., Zhu X., Huang K. (2021). Early 2 factor (E2F) transcription factors contribute to malignant progression and have clinical prognostic value in lower-grade glioma. *Bioengineered*.

[3] Ye L., Xu Y., Wang L. (2021). Downregulation of CYP2E1 is associated with poor prognosis and tumor progression of gliomas. *Cancer Medicine*.

[4] Narita Y., Muragaki Y., Kagawa N. (2021). Safety and efficacy of depatuxizumab mafodotin in Japanese patients with malignant glioma: a nonrandomized, phase 1/2 trial. *Cancer Science*.

[5] Zhao Z., Wu Y., Wang Z., Xu J., Wang Y., Zhao Z. (2021). Establishment and validation of five autophagy-related signatures for predicting survival and immune microenvironment in glioma. *Genes & Genomics*.

[6] Bauman M. M. J., Bhandarkar A. R., Zheng C. R. (2021). Management strategies for pediatric patients with tectal gliomas: a systematic review. *Neurosurgical Review*.

[7] Fukui A., Muragaki Y., Saito T. (2021). Impact of awake mapping on overall survival and extent of resection in patients with adult diffuse gliomas within or near eloquent areas: a retrospective propensity score-matched analysis of awake craniotomy vs. general anesthesia. *Acta Neurochirurgica*.

[8] Chakrabarti R., Gupta V., Vyas S., Gupta K., Singh V. (2021). Correlation of dual energy computed tomography electron density measurements with cerebral glioma grade. *The Neuroradiology Journal*.

[9] Manju C. A., Jeena K., Ramachandran R. (2021). Intracranially injectable multi-siRNA nanomedicine for the inhibition of glioma stem cells. *Neuro-Oncology Advances*.

[10] Chen X., Li C., Li Y. (2021). Characterization of METTL7B to evaluate TME and predict prognosis by integrative analysis of multi-omics data in glioma. *Frontiers in Molecular Biosciences*.

[11] Wang F., Dong L., Wei X. (2021). Effect of gambogic acid-loaded porous-lipid/PLGA microbubbles in combination with ultrasound-triggered microbubble destruction on human glioma. *Frontiers in Bioengineering and Biotechnology*.

[12] Qi Z. Y., Wang L. L., Qu X. L. (2021). lncRNA LINC00355 acts as a novel biomarker and promotes glioma biological activities via the regulation of miR-1225/FNDC3B. *Disease Markers*.

[13] Lv Z., Qiao L. (2020). Analysis of healthcare big data. *Future Generation Computer Systems*.

[14] Rayi A., Alnahhas I., Ong S., Giglio P., Puduvalli V. K. (2021). Targeted therapy for BRAF mutant brain tumors. *Current Treatment Options in Oncology*.

[15] Giovagnoli A. R., Meneses R. F., Paterlini C., Silvani A., Boiardi A. (2021). Cognitive awareness after treatment for high-grade glioma. *Clinical Neurology and Neurosurgery*.

[16] Raj D., Agrawal P., Gaitsch H., Wicks E., Tyler B. (2021). Pharmacological strategies for improving the prognosis of glioblastoma. *Expert Opinion on Pharmacotherapy*.

[17] Shen C. J., Terezakis S. A. (2021). The evolving role of radiotherapy for pediatric cancers with advancements in molecular tumor characterization and targeted therapies. *Frontiers in Oncology*.

[18] Zhong G., Wang Y., Wang Q. (2021). Discovery of novel ID2 antagonists from pharmacophore-based virtual screening as potential therapeutics for glioma. *Bioorganic & Medicinal Chemistry*.

[19] Lin W. W., Ou G. Y., Zhao W. J. (2021). Mutational profiling of low‐grade gliomas identifies prognosis and immunotherapy‐related biomarkers and tumour immune microenvironment characteristics. *Journal of Cellular and Molecular Medicine*.

[20] Li G., Zhang Z., Cai L. (2021). Fn14-targeted BiTE and CAR-T cells demonstrate potent preclinical activity against glioblastoma. *OncoImmunology*.

[21] Tian F.-X., Ma H.-F., Zhang Q. (2020). Identification of mir-9 in glioma diagnosis and prognosis. *Clinical Laboratory*.

[22] Higuchi F., Nagashima H., Ning J., Koerner M. V. A., Wakimoto H., Cahill D. P. (2020). Restoration of temozolomide sensitivity by PARP inhibitors in mismatch repair deficient glioblastoma is independent of base excision repair. *Clinical Cancer Research*.

[23] Bai H.-L., Kang C.-M., Sun Z.-Q. (2020). TTDA inhibited apoptosis by regulating the p53-Bax/Bcl2 axis in glioma. *Experimental Neurology*.

[24] Kumar A., Chandra P., Kale S. (2020). Parietal transventricular approach for medial temporal glioma: a technical report. *Surgical Neurology International*.

[25] Chan H.-M., Loh W. N.-H., Yeo T. T., Teo K. (2019). Awake craniotomy and excision of a diffuse low-grade glioma in a multilingual patient: neuropsychology and language. *World Neurosurgery*.

[26] Xin S., Huang K., Zhu X.-G. (2019). Non-coding RNAs: regulators of glioma cell epithelial-mesenchymal transformation. *Pathology, Research & Practice*.

[27] Jenkinson M. D., Barone D. G., Bryant A. (2018). Intraoperative imaging technology to maximise extent of resection for glioma. *Cochrane Database of Systematic Reviews*.

[28] Fujita Y., Kohta M., Sasayama T. (2020). Intraoperative 3-T magnetic resonance spectroscopy for detection of proliferative remnants of glioma. *World Neurosurgery*.

[29] Yu Z., Amin S. U., Alhussein M., Lv Z. (2021). Research on disease prediction based on improved DeepFM and IoMT. *IEEE Access*.

[30] Pichierri A., Bradley M., Iyer V. (2019). Intraoperative magnetic resonance imaging-guided glioma resections in awake or asleep settings and feasibility in the context of a public health system. *World Neurosurgery: XL*.

[31] Xie S. X., Yu Z. C., Lv Z. H. (2021). Computer modeling in engineering & sciences. *Tech SciencePress*.

[32] Chen Z.-J., Zheng J.-L., Guan W., Liu W.-G., Zheng J.-Y., Zuo J.-D. (2019). Intraoperative perfusion-weighted imaging in non-enhanced glioma surgery. *Minerva Chirurgica*.

[33] Kondo A., Akiyama O., Aoki S., Arai H. (2020). Application of intra-operative magnetic resonance imaging for intracranial epidermoid cysts. *British Journal of Neurosurgery*.

